# Development and evaluation of a point-of-care ocular ultrasound curriculum for medical students - a proof-of-concept study

**DOI:** 10.1186/s12909-023-04723-1

**Published:** 2023-10-03

**Authors:** Johannes Matthias Weimer, Maximilian Rink, Thomas Vieth, Jonas Lauff, Andreas Weimer, Lukas Müller, Marie Stäuber, Sebastian R. Reder, Holger Buggenhagen, Henrik Bellhäuser, Roman Kloeckner, Julian Künzel, Esther M. Hoffmann, Anna Würde

**Affiliations:** 1 Rudolf Frey Learning Clinic, University Medical Center of the Johannes Gutenberg University Mainz, Mainz, Germany; 2 Department of Otorhinolaryngology, Head and Neck Surgery, University Hospital Regensburg, Regensburg, Germany; 3 Center of Orthopedics, Trauma Surgery, and Spinal Cord Injury, Heidelberg University Hospital Heidelberg, Heidelberg, Germany; 4 Department of Diagnostic and Interventional Radiology, University Medical Center of the Johannes Gutenberg University Mainz, Mainz, Germany; 5 Department of Anesthesia, Intensive Care Medicine, Emergency medicine, Pain medicine, University Medical Center of the Johannes Gutenberg University Mainz, Mainz, Germany; 6 Department of Neuroradiology, University Medical Center of the Johannes Gutenberg University Mainz, Mainz, Germany; 7 Institute of Psychology, Johannes Gutenberg University of Mainz, Mainz, Germany; 8 Institute of Interventional Radiology, University Hospital Schleswig-Holstein - Campus Lübeck, Lübeck, Germany; 9 Department of Ophthalmology, University Medical Center of the Johannes Gutenberg- University Mainz, Mainz, Germany

**Keywords:** Point-of-care-sonography, POCUS, Ocular ultrasound, POCOUS, Ultrasound training, Curriculum development, Imaging, Sonography, Ultrasound training, Blended learning, Eye sonography

## Abstract

**Background:**

Point-of-care Ocular Ultrasound (POCOUS) has gained importance in emergency medicine and intensive care in recent years. This work aimed to establish and evaluate a dedicated ultrasound education program for learning POCOUS-specific skills during medical studies at a university hospital.

**Methods:**

The blended learning-based program (6 teaching units) based on recent scientific publications and recommendations was developed for students in the clinical part of their medical studies. Experts and trainers consisted of physicians from the Ear-Nose-Throat, radiology, ophthalmology and neurology specialties as well as university educational specialists. Lecture notes containing digital video links for preparation was produced as teaching material. In total, 33 students participated in the study. The education program, including the teaching materials, motivation and subjective gain in competency, was evaluated with the aid of a questionnaire (7-point Likert response format). Objective learning success was assessed on the basis of pre- and post-tests. These covered the skill areas: “anatomical basics”, “ultrasound basics”, “understanding of cross-sectional images”, “normal findings” and “pathology recognition”.

**Results:**

In the objective assessment of image interpretation, the participants improved significantly (p < 0.001) from pre- to post-test with a large effect size (Cohen’s d = 1.78, effect size r = 0.66). The evaluations revealed a high level of satisfaction with the course concept, teaching materials and the tutors. In addition, a high level of motivation was recorded in relation to continuing to study “ultrasound diagnostics” and “ophthalmologic diseases”. A significant (p < 0.01) positive gain was also achieved in terms of the subjective assessment of competency. This covers areas such as expertise, sonographic anatomy and performing a POCOUS examination as well as recognizing retinal detachment, globe perforation and increased optic nerve sheath diameter.

**Conclusion:**

The results of this feasibility study show that medical students accept and support a POCOUS-specific education program and are able to develop a higher objective and subjective level of competency. Future transfer to other sites and larger groups of participants seems feasible.

**Supplementary Information:**

The online version contains supplementary material available at 10.1186/s12909-023-04723-1.

## Introduction

### Background

Medical education is focusing increasingly on practice-oriented training and the associated development of skills of interdisciplinary importance. Ultrasound diagnostics meets the criteria for skills-based interdisciplinary teaching almost perfectly and education programs for this diagnostic modality are welcomed by students and perceived as enriching their training [1–7]. The recently published programs focus on teaching ultrasound skills in the field of abdominal ultrasonography, echocardiography and emergency ultrasonography, which includes point-of-care ultrasonography (POCUS) [2, 3, 6, 8–15]. Increasing use is being made of “blended learning” as a teaching method [16–19]. This term is used to define pedagogical approaches, which combine traditional in-person learning with digital teaching formats and transfer work on key theoretical content to a preparatory phase [18, 20]. Ultrasound diagnostic education in the fields of ophthalmology, neurology and ear, nose and throat medicine during medical studies has only been investigated in a few studies so far [8, 10, 12, 13, 21]. However, there are some unique factors predisposing ocular ultrasound diagnostics for incorporation into student education:

1. As a superficially located, fluid-filled organ, the eye lends itself well to visualization by means of ultrasonography.
2. The examination is non-invasive and does not involve exposure to radiation. Mutual training of students is possible if certain safety aspects are observed.
3. Often pre-existing knowledge of ultrasonography allows for a steep learning curve [22].
4. The approach can also be implemented by physicians from other specialties who do not work in ophthalmology and used for specific questions, for example in neurology, intensive care and emergency medicine [21–31].

A combination of examination methods is advisable with regard to the specific clinical issues, individual examiner skill and availability of diagnostic tools [32].

### Research problem & aim

Ultrasonography increasingly becoming part of the med school curriculum and is mentioned in several learning objectives for various specialties in the current “National Competence-Based Learning Objective Catalogue of Medicine” (NKLM) and several international learning recommendations [12, 33, 34]. In the case of “retinal detachment”, for example, reference in the NKLM is made via the associated learning objectives to the selection of ultrasonography as an instrument-based method [33]. This prospective proof-of-concept study aimed therefore to evaluate the development and implementation of a blended learning-based education program designed for students in the field of POCOUS at a university hospital. Furthermore, this study aimed to investigate the potential of the associated innovative teaching methods to impart specific skills and increase students’ interest in a certain medical specialty.

## Materials and methods

### Study description

**Fig. 1:**
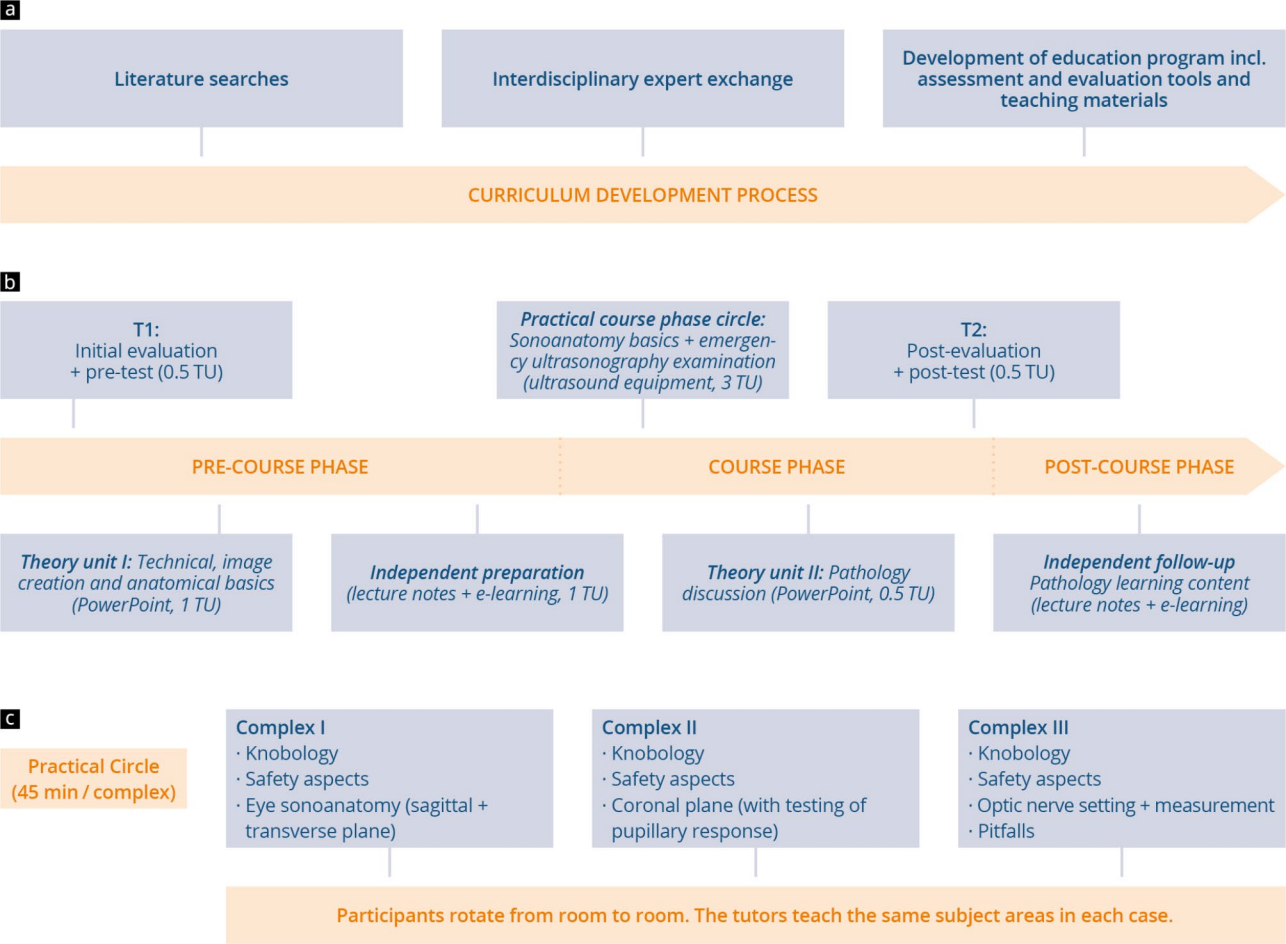
Representation of the point-of-care ocular ultrasound curriculum **a**) POCOUS curriculum development process; **b**) chronological representation of the whole curriculum and study procedure including data collection times (T1 and T2); **c**) Circle of the practical course phase with three complexes

This feasibility study was planned prospectively at the Learning Clinic and the Department of Ophthalmology and Radiology at our University Medical Center. Its implementation complied with the “Strengthening the Reporting of Observational Studies in Epidemiology” (STROBE) criteria [35]. Written evaluations and theoretical learning outcomes assessments were carried out at two time points (T1 and T2) in order to measure subjective and objective acquisition of skills and acceptance of the training curriculum [36]. Figure 1 visualizes the study procedure and the course model. The primary goal of the study was to evaluate the subjective as well as objective competence gain of the participants. Secondarily, the acceptance of the concept and the intrinsic motivation of the participants was to be determined. Inclusion criteria were passing the 1st state exam and full participation in the entire course concept, including the examinations.

### Material

#### Training model and learning objectives

The curriculum was developed bearing in mind recommendations of professional associations, recent publications and (international) university approaches to ultrasound training [8, 10, 21, 22, 30, 31, 37, 38]. Interdisciplinary exchanges between ophthalmology, neurology, radiology and otorhinolaryngology also took place for this purpose to take into account the relevant issues of the different disciplines. The curriculum including pre- and post-test comprised 6 teaching units (TU) (45-minutes each) and was divided into a pre-course, in-person course and post-course phase in accordance with the “blended learning” [17, 18] approach to teaching (see Fig. 1). The defined theoretical and practical learning objectives are listed in Table 1, the focus being on teaching “technical and equipment basics”, “physiological anatomical knowledge”, the “sonomorphology of normal findings”, “measurement of optic nerve sheath diameter and pupillary response” and recognizing “pathologies of relevance to emergency medicine”. Within the course, the following pathologies were discussed in the lecture notes as well as in the second theory unit using exemplary images: Retinal detachment (amotio retinae), vitreous hemorrhage, intraocular foreign body and penetrating bulb injury, lens dislocation, tumors of the eye, endophthalmitis, increased intracranial pressure with elevation of ONSD. Special attention was given to the ultrasound appearance of these pathologies. Treatment was not subject of the course.

**Table 1:** Theoretical and practical learning objectives of the ocular ultrasound curriculum

Theoretical learning objectives	Practical learning objectives
• Safety aspects • Ultrasound machine design including knobology • Transducer types • Technical and equipment basics • Anatomy of the eye • Understanding of ultrasound orientation views/sections • Ocular ultrasound examination procedure including pitfall awareness • Ultrasound-assisted pupillary response testing • Placement of optic nerve sheath diameter (ONSD) measurement in clinical context • Options for documenting findings • Recognizing retinal detachment, vitreous detachment, vitreous hemorrhage, lens dislocation, foreign bodies and space-occupying lesions on the ultrasound image and differentiating between them	• Machine setup (transducer selection, preset selection, image optimization) • Mastering transducer handling (holding, movements, stabilization, connection) • Drawing ultrasound orientation views • Adjusting ocular ultrasound orientation views • Ultrasound assessment of the eye in 2 cross-sectional planes • Testing pupillary response using ultrasound • ONSD measurement • Documentation of findings recorded on the ultrasound machine

### Preparatory phase/pre-course phase

In this phase, the participants took part in a plenary in-person introductory session. This started (T1) with a written evaluation and pre-test (0.5 TU). The participants then received education in theory (theory unit I) via screen presentation (1 TU) and lecture notesincluding links to online videos on a video portal and teaching websites (examination procedures and normal findings) via QR code (for excerpt, see Supplement Fig. 1) The participants were supposed to work with these materials (scheduled time 1 TU) on their own and prepare for the face-to-face course phase. The time between the introductory session and the start of the face-to-face course was 1 week.

### Course phase

During the in-person course phase (see Fig. 1c), the participants received instruction on the ultrasound equipment in a practice circle (three stations with in total 3TU) in groups of four [1, 3]. During the course the students practice the ultrasound examinations on each other. A total of 8 ultrasound machines from GE HealthCare (three GE F8 and two GE VSCAN extend; General Electric Company, Boston) and Philips (three HD 5; Philips GmbH, Eindhoven), each with high-frequency linear transducers and presets specially configured for the course, were used for the practical exercises. Integrated into the course phase was a presentation of selected pathologies (theory unit II) based on case studies and exemplary ultrasound images (0,5 TU). The course phase ended with an evaluation and post-test (0.5 TU) (T2).

### Follow-up phase/post-course phase

After completing the in-person part of the course, the participants had the opportunity to deepen and consolidate their knowledge of the content again with the aid of the lecture notes, including the supplements available online. The participants also received a motivational email one week after the course to encourage them to do this.

### Teaching material

For the in-person course phase preparation and follow-up, the participants were provided with dedicated lecture notes. This illustrated basic (sono-)anatomy, the clinical relevance of the method and POCOUS with relevant images, ultrasound clips and explanatory videos (accessible via QR codes) as well as additional online resources. Learning outcomes could be assessed individually by means of check lists (for excerpt of the lecture notes, see Supplement Fig. 1).

#### Lecturers and tutors

The lecturers and tutors were specialists (2x), residents (2x) and last year medical students (2x) who received additional education in advance and digital briefing (4 teaching units) and had already performed > 40 ocular ultrasound scans.

### Methods

#### Recruitment of participants

The study participants were medical students starting from the third year who had applied voluntarily via an internet portal. The corresponding students received information about the course and the opportunity to apply via an email from the study office sent out centrally. The students were not advantaged or disadvantaged in compulsory curriculum courses as a result of their decision for or against participation.

### Evaluation form and learning outcomes assessment

To measure skills acquisition and attitude to the curriculum, written evaluations and learning outcomes assessments were carried out at two time points (T1 + T2). The evaluation forms (see Supplementary Fig. 2a + b)asked questions about the main areas, “prior knowledge”, “expectations and needs”, “interest and motivation”, “attitude to ultrasound teaching”, “competency assessment” and “satisfaction with the curriculum, teaching materials and tutors” with the aid of various subitems. Dichotomous (yes/no) questions, free-text questions and questions with a seven-point Likert scale (1 = fully agree; 7 = do not agree at all) were used for this. The learning outcomes assessments (max. 50 points) included assessment of “anatomical basics” (max. 9 points), “ultrasound basics” (max. 16 points), “cross-sectional imaging skills” (max. 10 points), “normal findings + measured values” (max. 9 points) and “pathologies” (max. 6 points) on the basis of free-text tasks [39] (for sample tasks, see Supplement Fig. 3). The pre-test and post-test questions covered the same content with partially different images.

### Statistics

The data from the written evaluation forms and learning outcomes assessments were saved with Microsoft Excel. All statistical analyses were performed in RStudio (RStudio Team [2020]. RStudio: Integrated Development for R. RStudio, PBC, http://www.rstudio.com, last accessed on 15 04 2023) with R 4.0.3 (A Language and Environment for Statistical Computing, R Foundation for Statistical Computing, http://www.R-project.org; last accessed on 15 06 2022). Binary and categorical baseline variables are given as absolute numbers and percentages. Continuous data are given as median and interquartile range (IQR) or as mean and standard deviation (SD). Categorical variables were compared using Fisher’s exact test and continuous variables using the Mann-Whitney U test. In addition, a multivariate linear regression model was produced in order to compare the influence of individual factors. P-values < 0.05 were considered statistically significant.

## Results

### Study population

In total, n = 33 participants were included in the study. The baseline characteristics of the study group are listed in Table 2. All participants had already passed the first state exam and in some cases (36.4%) had previous professional education (e.g. Nursing). Most of the participants were female (60.6%) and at the time of the course were in the first half of the clinical part of their medical studies (semester 5–7 = 81.8%). More than half of the participants had already completed their clinical studies in ophthalmology, ENT, and neurology (> 50%) and had previous experience in the field of ultrasound diagnostics (> 80%). Nevertheless, almost all participants (97%) stated that they had not yet come into contact with ocular ultrasonography.

**Table 2:** Baseline characteristics of participants (N = 33)

Mean age ± SD	26 ± 5
**Gender n (percentage)**	
Female Male	20 (61) 13 (39)
**Stage of studies n (percentage)**	
Semester 5 Semester 6 Semester 7 Semester 8 Semester 9 Semester 10 Last year (Semester 11 + 12)	11 (33) 8 (24) 8 (24) 3 (9) 2 (6) 0 (0) 1 (3)
**Prior training n (percentage)**	
Yes No	12 (36) 21 (64)
University training subjects already completed* **n (percentage)**	
Ophthalmology Neurology Neurosurgery ENT (Ear-Nose-Throat)	19 (58) 21 (64) 1 (3) 21 (64)
**Clerkship n (percentage)**	
Ophthalmology Neurology Neurosurgery ENT (Ear-Nose-Throat) Emergency department Intensive care none	2 (6) 4 (12) 0 (0) 0 (0) 2 (6) 0 (0) 25 (76)
**Previous experience in ocular ultrasonography**	
Yes No	1 (3) 32 (97)
Other ultrasound experience*	
Weekly course abdomen (15 h) Weekend course abdomen (15 h) Cardiac ultrasonography course (9 h)	28 (85) 5 (15) 19 (58)

* the summed percentage value can be > 100%, as the participants may have attended several courses

### Subjective results/survey results

The results of the statements asked about on the subjects “interest”, “course motivation”, “course follow-up” and “attitude” can be found in Supplement Table 1. Interest in ophthalmology in particular showed the highest gain in scale points tendentially compared with the other subjects asked about (T1 mean 3.00, SD [1.92] vs. T2 mean 2.41, SD [1.55]; p = 0.27).

“General” course motivation was scored excellent by the participants (mean 1.55, SD [0.754]), while in both the T1 and T2 assessments the motivation for “ultrasound diagnostics” (T1 mean 1.27, SD [0.45]) and “Gain in-depth ultrasound knowledge” (T2 mean 1.37, SD [0.57]) as well as “ophthalmology” (T1 mean 1.76, SD [1.20] and “Gain in-depth ophthalmologic diseases” (T2 mean 1.81, SD [1.24]) was rated highest by the participants (see also Supplement Table 1). Furthermore, the participants stated that they wished to continue studying ultrasonography after the event (T2 mean 1.19, SD [0.40]) and will also use the teaching material used in the course for this (T2 mean 1.85, SD [0.95]).

The items asked about in relation to “attitude to ultrasound teaching” (< 1.3 scale points), “ultrasound teaching media” (< 2.5 scale points) and “teaching generally” (< 1.8 scale points) were consistently rated at the high end of scale ranges during the course, with the highest increase in agreement tendentially being recorded for the items “more efficient learning with digital than with purely analog teaching media” (T1 mean 2.48, SD [1.56] vs. T2 mean 2.07, SD [1.14]; p = 0.42) and “influence of teaching quality in the specialty of choice of specialist medical training” (T1 mean 1.73, SD [1.23] vs. T2 mean 1.41, SD [0.64]; p = 0.39).

The results for the participants’ subjective development of competency are presented in Table 3. A significant gain in competency was measured here in all items asked about under “general ultrasound skills” and “eye-specific skills” (p < 0.01). No significant gain in competency was measured within “skills in associated areas” (Slit lamp examination, Ophthalmoscopy, Assessment of intracranial pressure with cross-sectional imaging, Clinical neurological examination).

**Table 3:** Participants’ subjective (development of) competency

	T1	T2	Effect size	p-value
	Mean ± SD	Mean ± SD	Cohens d	
**General ultrasound skills (1 = very high; 7 = very low)**				
Theoretical knowledge	5.24 ± 1.70	2.19 ± 0.56	2.33	< 0.01
Equipment use	4.61 ± 1.78	2.07 ± 0.68	1.81	
Transducer handling	4.48 ± 1.73	1.89 ± 0.64	1.91	
Spatial orientation	4.94 ± 1.64	2.11 ± 0.93	2.07	
Sonoanatomical assignment	5.39 ± 1.64	1.70 ± 0.72	2.82	
Organ visualization	5.21 ± 1.71	2.00 ± 0.78	2.34	
Organ assessment	5.45 ± 1.64	2.44 ± 0.89	2.22	
Patient guidance	4.70 ± 1.78	1.81 ± 0.74	2.05	
Safety aspects	6.06 ± 1.43	1.81 ± 0.92	3.45	
Understanding of pathology	6.33 ± 1.08	3.37 ± 1.15	2.67	
**Eye-specific skills (1 = very high; 7 = very low)**				
Ocular ultrasound assessment	6.03 ± 1.65	2.41 ± 1.37	2.37	< 0.01
Ultrasound assessment of pupillary response	6.24 ± 1.30	2.30 ± 1.71	2.64	
Measurement of ONSD	6.42 ± 1.28	2.78 ± 1.65	2.51	
Ultrasound identification of retinal detachment	6.24 ± 1.28	2.39 ± 1.94	2.06	
Ultrasound identification of globe perforation/penetration	6.18 ± 1.40	3.63 ± 1.62	1.70	
**Skills in associated areas (1 = very high; 7 = very low)**				
Slit lamp examination	5.39 ± 1.68	5.15 ± 1.73	0.14	0.54
Ophthalmoscopy	5.45 ± 1.54	4.70 ± 1.73	0.46	0.10
Assessment of intracranial pressure with cross-sectional imaging (cranial computed tomography = cCT/cranial magnetic resonance imaging = cMRI)	5.67 ± 1.80	5.04 ± 1.95	0.34	0.20
Clinical neurological examination	4.58 ± 1.95	4.26 ± 1.79	0.17	0.46

Supplement Table 2 shows the course evaluation results in relation to the “course concept”, “teaching materials”, “tutors”, “ultrasound equipment” and “theory quizzes”. All the items asked about here (apart from the “theory quizzes” and “ultrasound instructional videos”) were rated at the high end of scale ranges (< 1.6 SP). The “clarity and structure of the course concept” (T2 mean 1.33, SD [0.48]), the ultrasound lecture notes with QR codes (T2 mean 1.30, SD [0.47]) and the technical (T2 mean 1.19, SD [0.40]) and teaching (T2 mean 1.22, SD [0.42]) skills of the tutors were rated particularly highly.

### Objective results/results of theory tests

**Fig. 2:**
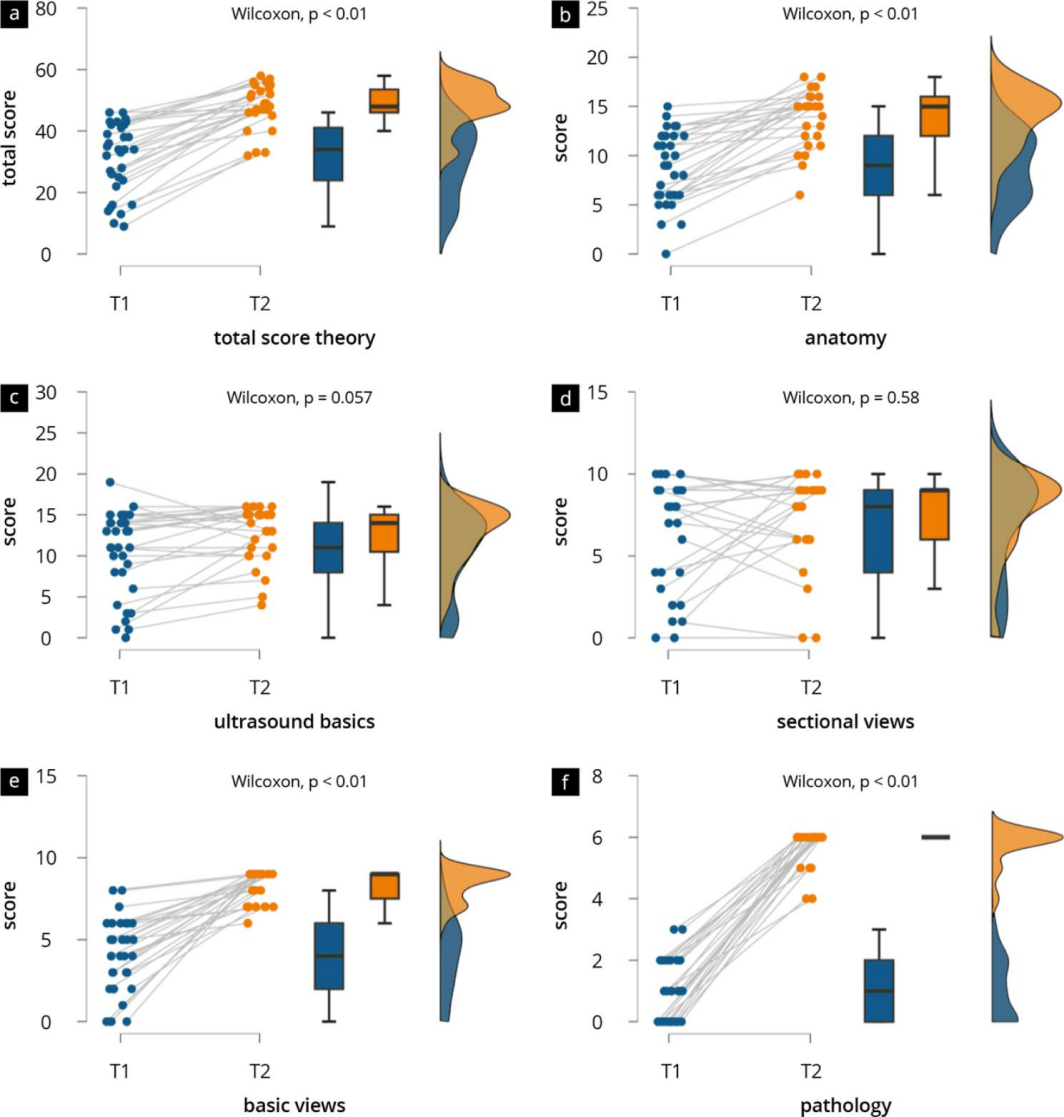
Results of theory test at time points T1-T2 by Raincloud Plots (**a**) total score; (**b**) anatomical basics; (**c**) ultrasound basics; (**d**) understanding of cross-sectional images; (**e**) normal findings; (**f**) pathology recognition

The results of the theory tests are presented in Fig. 2 and Supplement Table 3. Over the period from T1 to T2, participants achieved significantly higher results in terms of the total score (T1 mean 30.73, SD [11.33] vs. T2 mean 48.00, SD [7.25]; p < 0.01). With regard to the skill areas addressed in the testing, these results are reflected in “anatomical basics” (T1 mean 8.73, SD [3.65] vs. T2 mean 13.93, SD [2.96], “normal findings” (T1 mean 3.97, SD [2.27] vs. T2 mean 8.30, SD [0.95]), and “pathologies” (T1 mean 0.94, SD [1.00] vs. T2 mean 5.74, SD [0.59]. Although comparatively higher point scores were achieved in the pre-/post-test comparison in the skill areas “ultrasound basics” (T1 mean 10.36, SD [5.16] vs. T2 mean 12.63, SD [3.49]; p = 0.06) and “understanding of cross-sectional images” (T1 mean 6.72, SD [3.35] vs. T2 mean 7.41, SD [2.82]; p = 0.58), the results were not significant.

A correlation analysis revealed that the deltas of total objective test scores were not significantly correlated with the deltas of total subjective ratings (r=-0.071, p = 0.73). A comparison of the delta of the total objective scores of the tests (men 15.9 ± 7.2 vs. female 17.0 ± 9.1; p = 0.824) and the delta of the total subjective scores of the evaluations (men 3.6 ± 2.0 vs. female 3.5 ± 1.4; p = 0.920) showed no significant gender differences.

In the multivariate linear regression analysis in relation to the results of the theory tests at T1 and T2, previous ultrasonography experience (“weekly course abdomen”, “weekend course abdomen”, “cardiac ultrasonography course”), “Clerkships in ophthalmology” “prior training” and completion of “ophthalmology” as a subject within medical studies were defined as influencing factors, although in the overall T1 assessment only taking the “weekly course abdomen” (standardized regression coefficient β = 12.16; p = 0.04) had a significant influence. This also applies to the results of the “basics” theory test at T1 (β = 7.19. p < 0.01). In respect of the results for “normal findings”, attendance of the “weekend course” was found to be a significant influencing factor (β = 2.21; p = 0.049). With respect to the results for the T2 quiz, no significant influence was demonstrated for the defined factors.

## Discussion

The results of this study shows that the participants in the developed POCOUS curriculum were able to achieve an objectively measurable higher level of competency for this application. In addition, the curriculum was accepted exceptionally positively with regard to the course concept, teaching materials, tutors, ultrasound equipment and learning outcomes assessments. The digital teaching media/methods (“blended learning”) were not only accepted but there was a clear desire on the part of participants to develop these further. The motivation to continue studying ultrasonography and ophthalmology also increased. To our knowledge, a structured approach to teaching these aspects of POCOUS within the framework of medical studies has not existed previously.

### Development of competency

Participation in (peer-assisted) ultrasound training programs permits measurable subjective and objective development of competency [2, 6, 13, 15]. A significant subjective and objective increase in competency as a result of participation in our POCOUS curriculum was also demonstrated in this study.

In all the items in the categories “general ultrasound skills” and “eye-specific skills”, participants assessed their competency as significantly higher after the course. This also applies in particular to the recognition of retinal detachment and globe perforation or penetration. These selected conditions can also be encountered by physicians outside ophthalmology, e.g., in a general emergency department [30, 31, 40], and therefore represent an important learning objective within this POCOUS curriculum. No significant subjective increase in knowledge was identified in the category “skills in associated areas”. Because these subjects were not taught in the course, this shows that self-assessment in the post-test was not generally better and that there was no effect of social desirability in the answers.

An objective gain in knowledge was recorded in the categories: anatomical basics, normal findings and pathological findings. No significant improvement was found for the skill areas “ultrasound basics” and “understanding of cross-sectional images” in our work, which can be explained by the participants’ previous experience in relation to ultrasound courses already taken in other specialties (Table 2) [6, 41]. Overall, the results suggest that the course was successful in teaching the students the specific target skills. In order to determine practical skills, suitable examination platforms such as Direct Observation of Procedural Skills (DOPS) [36, 42] should be integrated into the curriculum in future. Furthermore not only the short-term gain in competency, but also long-term learning success are important aspects and should be reviewed in the future for the designed curriculum [43].

### Attitude to ultrasound teaching, acceptance of approach and future perspectives

Ultrasonography is increasingly being incorporated into various parts of medical education both nationally and internationally [1–3, 7, 10]. This applies in particular to courses in abdominal ultrasonography, FAST (focused assessment with sonography in trauma) and echocardiography [1–3, 6, 10, 12–14]. Little if any use is made of ultrasonography of the eyes and orbita, however [3, 8–10, 44]. International experts recommend teaching knowledge of ocular and optic nerve ultrasonography within medical studies curricula [34]. The study presented here is in line with this recommendation. Knowledge of selected pathologies associated with these structures is also taught in order to highlight possible clinical uses.

The integration of (extra-)curricular ultrasound-specific education programs is generally desired by students, despite the potentially additional workload [6, 7, 10, 41]. Our data also confirm a high level of satisfaction with the “concept” of the curriculum and student motivation to (continue) tackling ultrasound-specific learning content. Participants also expressed a desire for more teaching of this knowledge as part of the compulsory teaching program. As well as a generally high demand being indicated for teaching events on the subject of ultrasonography, a high level of interest existed among participating students in ophthalmology and ophthalmologic diseases in particular, as well as in continuing to study these subjects.

The high level of participant satisfaction with the lecturers and all tutors is remarkable. Besides a total of four physicians with varying degrees of experience, two students in their last year of medical studies were also selected for this. Because of their training status, the latter had slightly less ultrasound experience in a clinical context. Peer-to-peer teaching of the content of this course therefore seems to be very possible, if relevant intensive education is provided. This is in keeping with the results of other studies which have shown a high level of acceptance of well-trained student tutors [45]. This opens up the possibility of satisfying the desire for more practice-oriented teaching despite limited financial and human resources. With a peer-to-peer approach, smaller learning groups would be possible, which would increase the real hands-on time of performing ultrasound per student [13].

The participants in the POCOUS curriculum were in favor of increased use of digital teaching options, which are being used increasingly in student teaching [16, 46]. Future education programs should therefore be more heavily geared to blended learning [16, 18, 47–49]. This might make it possible to increase training time on the equipment during in-person phases and enhance the effectiveness of teaching by means of better participant preparation [49]. In addition, it would be desirable in future to develop and use models to simulate pathological findings which might positively influence the learning effect [40] and facilitate ultrasonography of these findings in practice for participants.

Overall, successful durable implementation of the POCOUS curriculum in future seems feasible with a limited commitment in terms of time and personnel [50]. The concept should be transferable to other sites. The recommendations of the NKLM [27] and the international expert consensus already mentioned [34] could therefore be met. Interdisciplinary exchange between different disciplines is also an important aspect of teaching ultrasound in a multidisciplinary way.

### Motivation and interest choice of specialist medical training

Many specialist medical disciplines have difficulty attracting new colleagues [51–53]. Besides compensation schemes during specialist medical training [51] or targeted mentoring programs within medical studies [53], innovative teaching programs are a method of inspiring lasting enthusiasm in a specialty among students. This POCOUS teaching program also enhanced interest in the specialties covered. This effect has also been observed as a result of teaching of other clinically practical skills [54, 55].

### Limitations

However, it is also important to be aware of the limitations of POCOUS (e.g. impossibility of completely ruling out eye disease requiring treatment) compared with an eye examination based on slit lamp examination and fundoscopy. Clinical ophthalmologic examination and ultrasonography should be selected depending on the clinical problem and availability and, if appropriate, should be used in a way that complements each other. This feasibility study was conducted with 33 participants. The participants may not therefore be completely representative of the entire student body because of the high level of previous experience with ultrasound and the high intrinsic motivation that can be assumed in the case of voluntary participation in a study. Assessing gains in competency on the basis of questionnaires and theory tests enables only limited conclusions to be drawn with regard to improvements in practical examination competency. Since we only conducted theoretical tests, we cannot make any statements about the development of the participants in the correct performance of the examination and image acquisition. Establishing appropriate testing formats for the POCOUS should be a target of future studies. In addition, no comparison group was used that only completed the two tests. A test effect bias can therefore not be completely excluded. Whether the improvement in competency achieved over the course of the study is permanent could be assessed with additional theory and practical assessments at later points in time. Another limitation is that no further subgroup analysis has been performed comparing the different semesters to another as the groups would have been too small for a well-powered analysis. Therefore, in terms of increasing participant interest, the results should be viewed critically. Future studies should further address whether the completion of ophthalmology during their studies did influence the test outcomes. A comparison with “traditional” teaching methods (without blended learning) was not made.

## Conclusion

The POCOUS curriculum presented here led to an increased competence of the participants. The course format and transfer of theoretical content to a (digital) preparatory phase was welcomed by the participants. It was possible for practical content to be taught by specially trained students with the possibility of supervision. For the future, the aim should be to incorporate the POCOUS curriculum into compulsory teaching in medical school. Integration into existing ultrasound teaching formats is possible with minimal adaptations. Complementary practical examinations such as DOPS should be developed to retrieve competencies in performing this diagnostic.

### Electronic supplementary material

Below is the link to the electronic supplementary material.

Supplementary Material 1

Supplementary Material 2

Supplementary Material 3

Supplementary Material 4

Supplementary Material 5

Supplementary Material 6

Supplementary Material 7

## Data Availability

Data cannot be shared publicly because of institutional and national data policy restrictions imposed by the Ethics committee since the data contain potentially identifying study participants’ information. Data are available upon request from the Johannes Gutenberg University Mainz Medical Center (contact via weimer@uni-mainz.de) for researchers who meet the criteria for access to confidential data (please provide the manuscript title with your enquiry).
