# Supplementary figures and images for: Development and evaluation of a point-of-care ocular ultrasound curriculum for medical students - a proof-of-concept study
Source: BMC Med Educ. 2023 Oct 3;23:723. doi: 10.1186/s12909-023-04723-1 (PMC10548604; doi:10.1186/s12909-023-04723-1)

**Supplementary figure 1** Excerpt of the course lecture notes

**
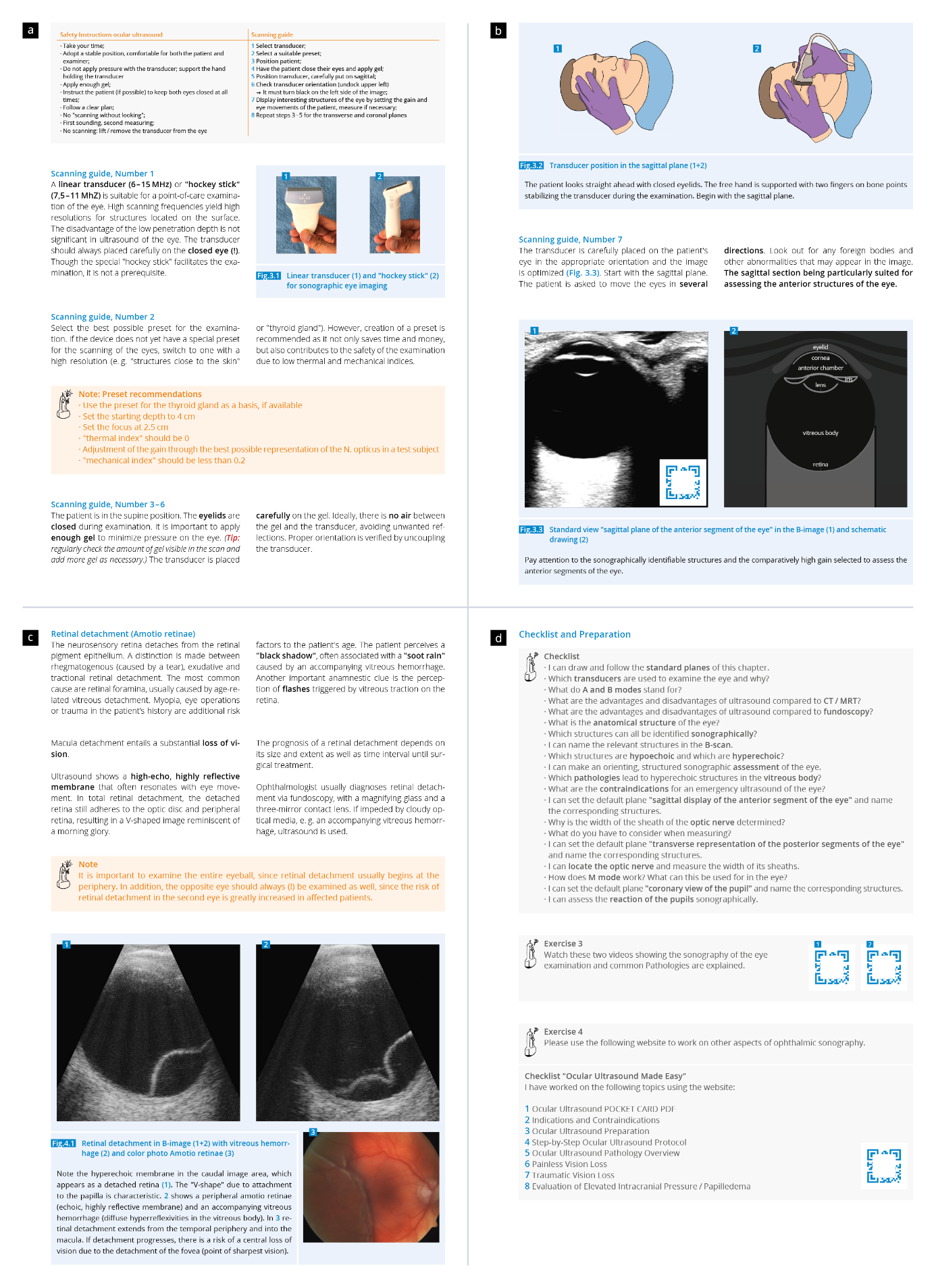
**

Supplement: Supplementary file 1 — Supplementary Material 1 [file 12909_2023_4723_MOESM1_ESM.docx]

**Supplementary figure 2a** Pre-evaluation form

**
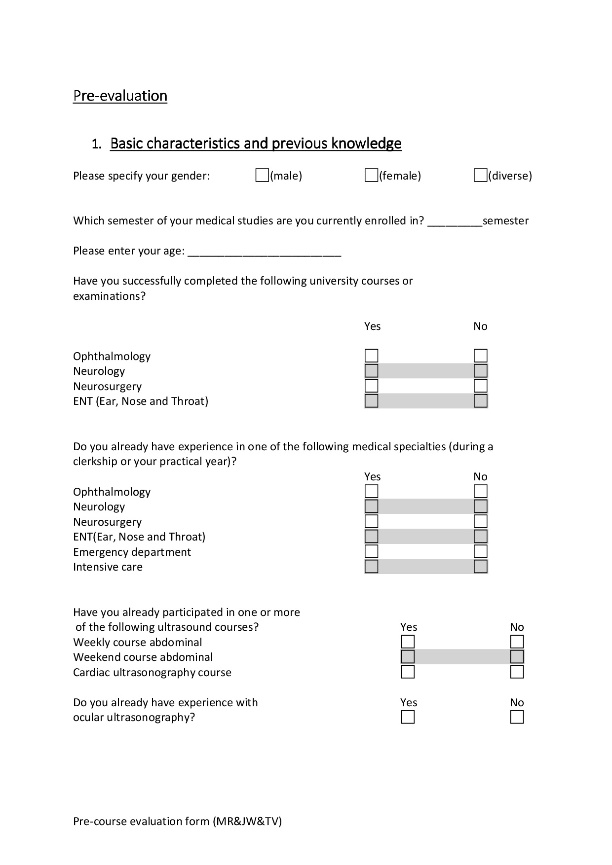

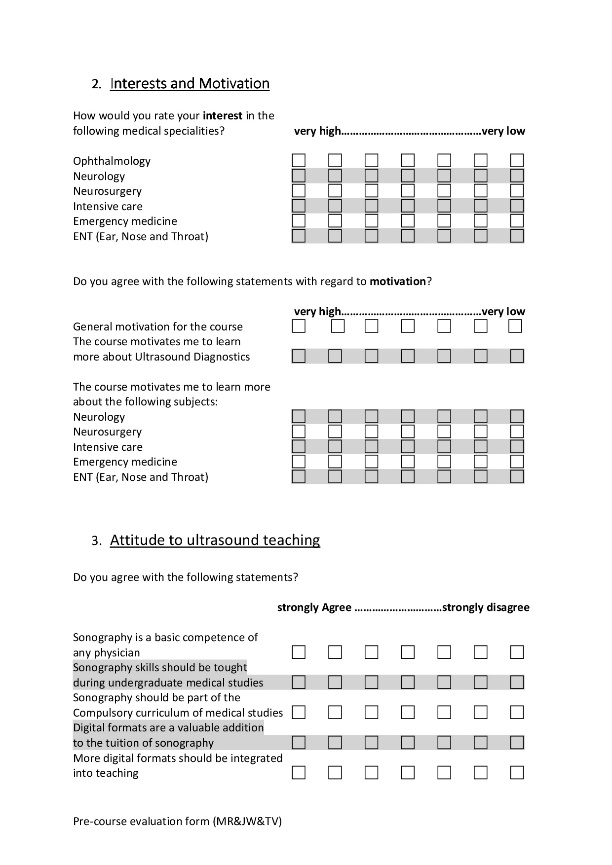
**

**
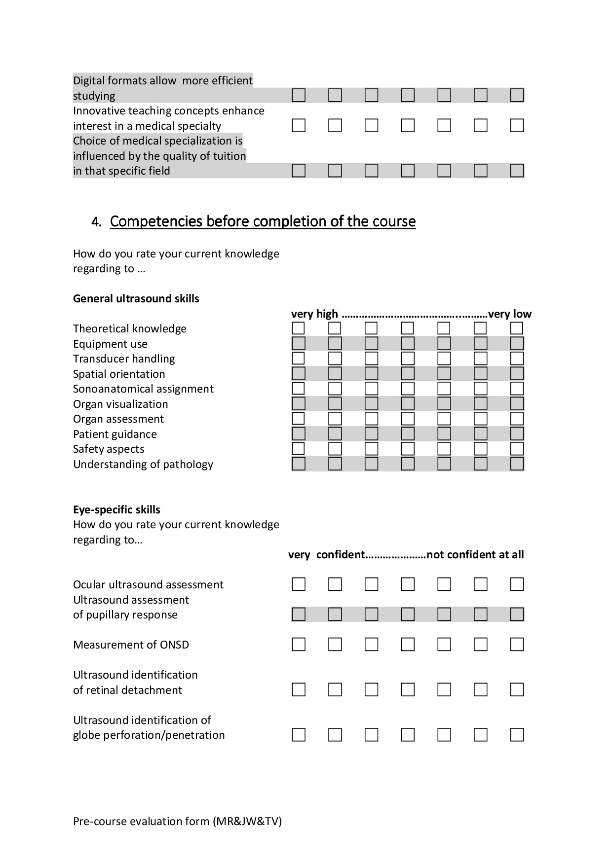

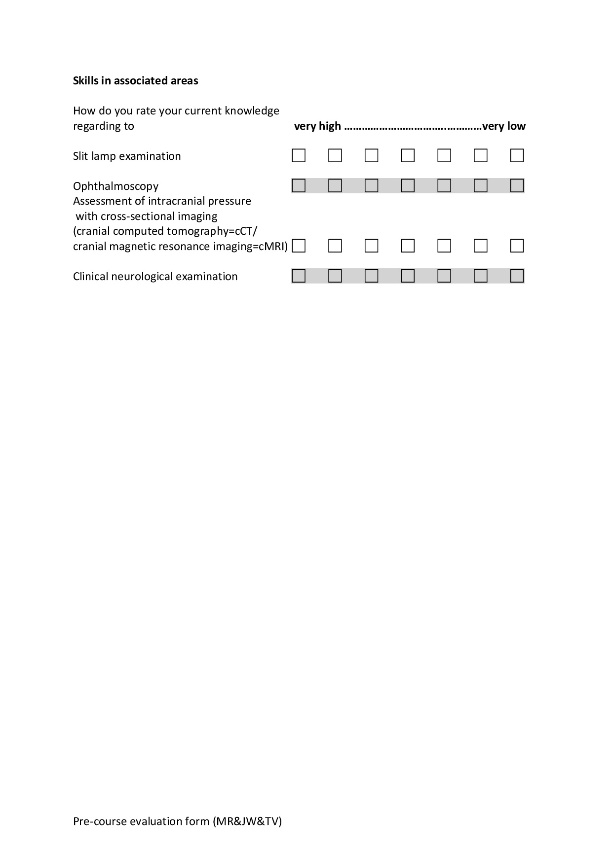
**

Supplement: Supplementary file 2 — Supplementary Material 2 [file 12909_2023_4723_MOESM2_ESM.docx]

**Supplementary figure 2b** Post-evaluation form

**
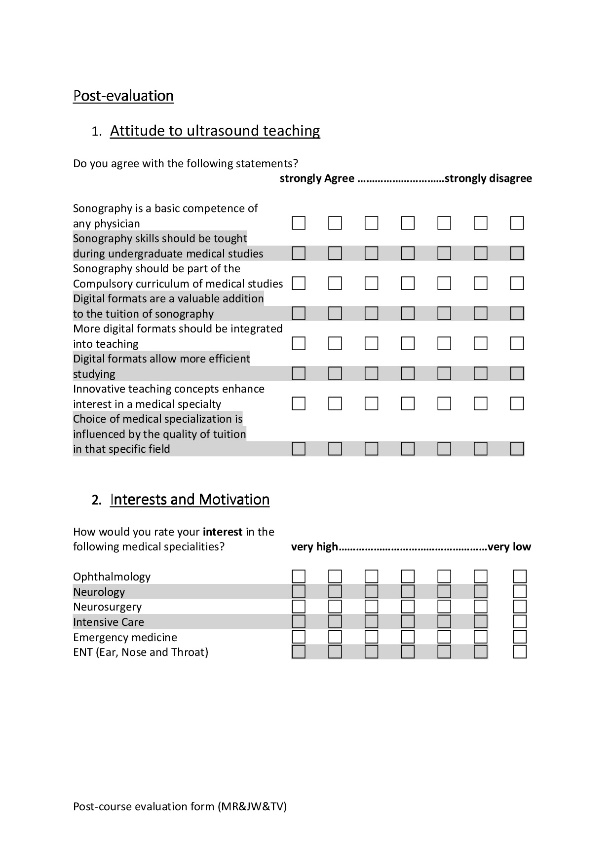

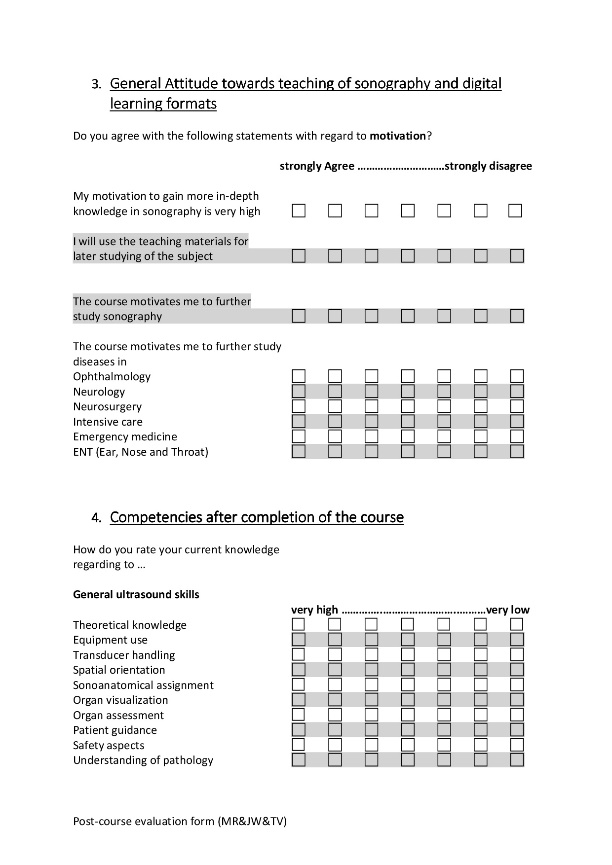
**

**
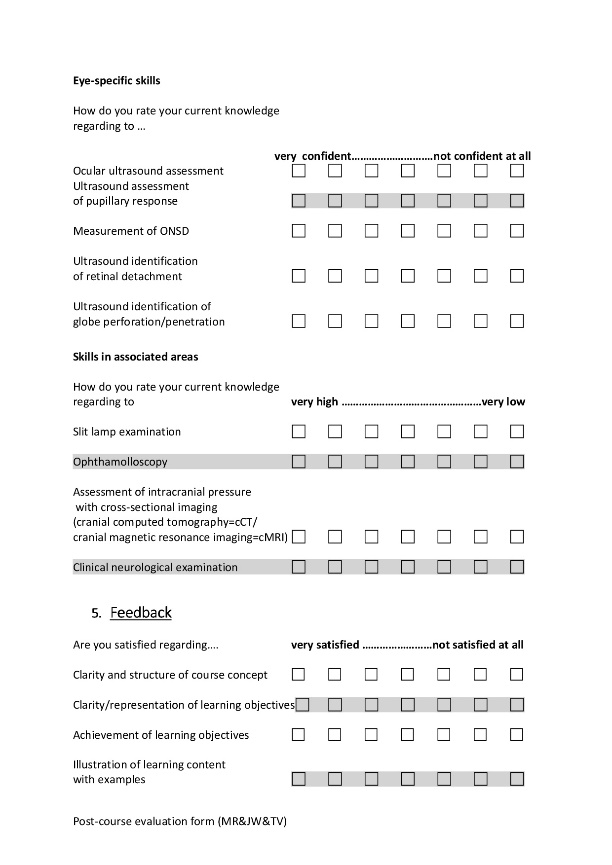

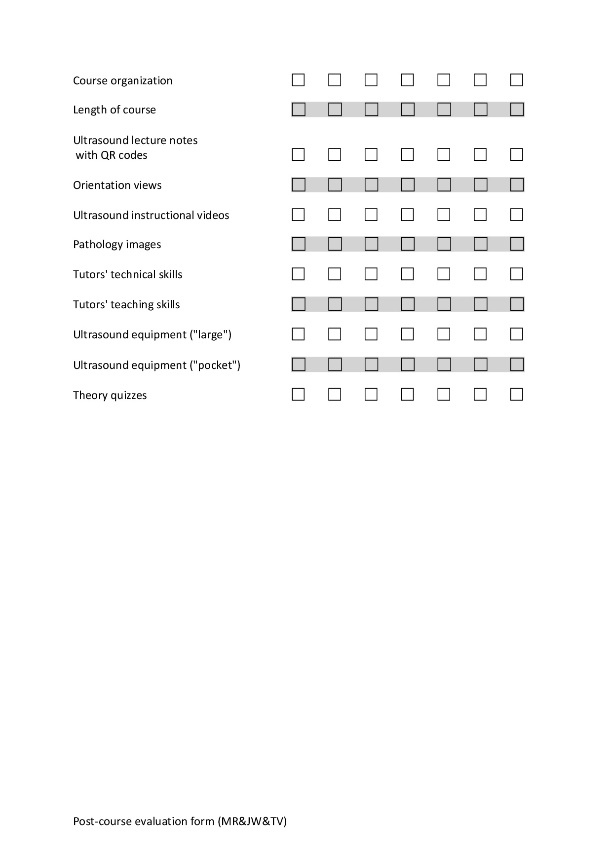
**

Supplement: Supplementary file 3 — Supplementary Material 3 [file 12909_2023_4723_MOESM3_ESM.docx]

**Supplementary figure 3** Excerpt of the theoretical test for competency measurement


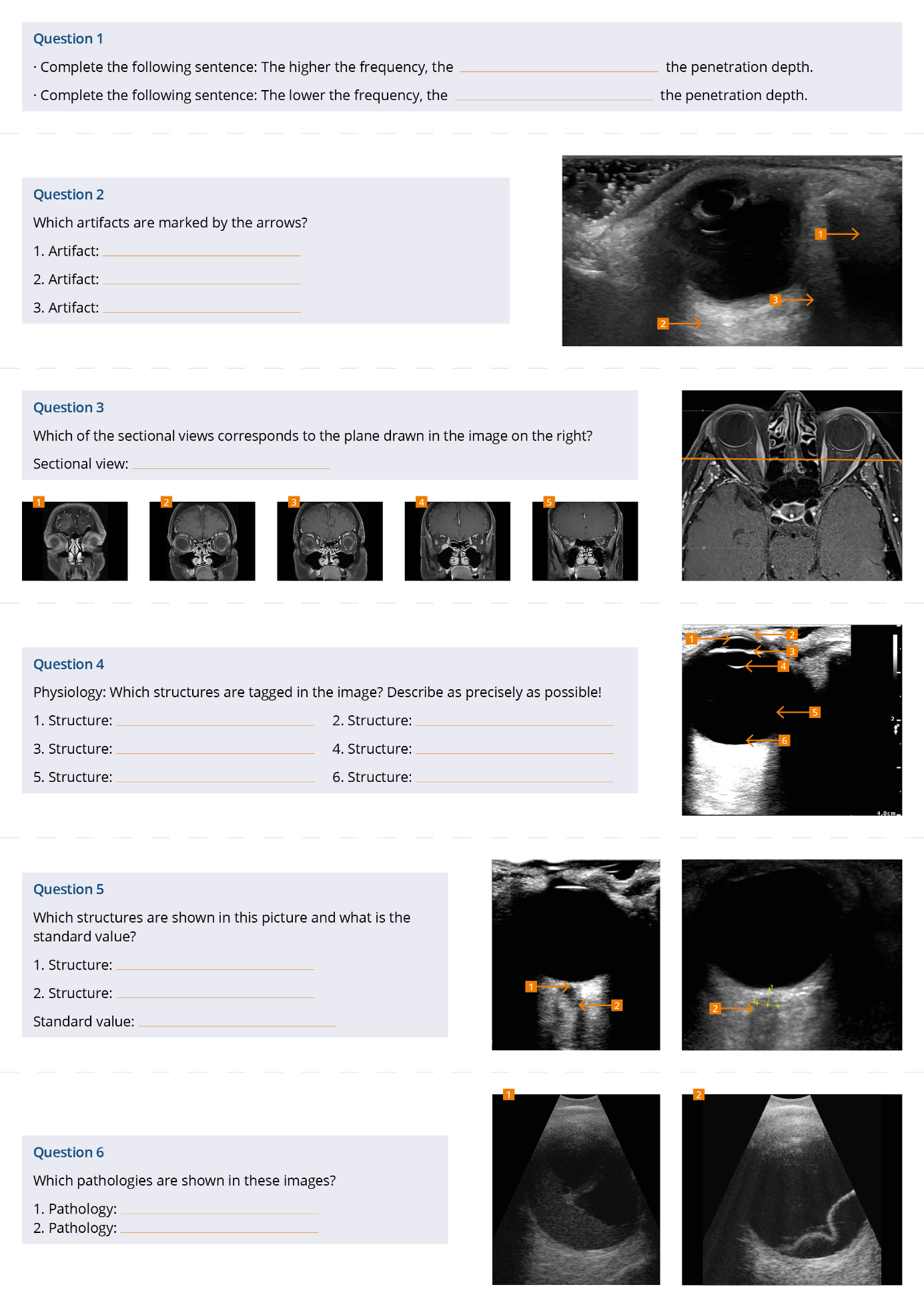

Supplement: Supplementary file 4 — Supplementary Material 4 [file 12909_2023_4723_MOESM4_ESM.docx]
