# Supplementary material for: Development and evaluation of a point-of-care ocular ultrasound curriculum for medical students - a proof-of-concept study
Source: BMC Med Educ. 2023 Oct 3;23:723. doi: 10.1186/s12909-023-04723-1 (PMC10548604; doi:10.1186/s12909-023-04723-1)
Supplement: Supplementary file 5 — Supplementary Material 5 [file 12909_2023_4723_MOESM5_ESM.docx]

**Supplementary Table 2** Participant course evaluation

|  | **T2** |
| --- | --- |
|  | **Mean ±SD** |
| **Course concept (1 = very high; 7 = very low)** | |
| Clarity and structure of course concept | 1.33 **±** 0.48 |
| Clarity/representation of learning objectives | 1.26 **±**0.44 |
| Achievement of learning objectives | 1.41 **±**0.50 |
| Illustration of learning content with examples | 1.33 **±**0.55 |
| Course organization | 1.59 **±**0.93 |
| Length of course | 1.41 **±**0.63 |
| **Teaching materials (1 = satisfaction very high; 7 = very low)** | |
| Ultrasound lecture notes  with QR codes | 1.30 **±**0.47 |
| Orientation views | 1.44 **±**0.58 |
| Ultrasound instructional videos | 2.04 **±**1.13 |
| Pathology images | 1.41 **±**0.50 |
| **Tutors (1 = satisfaction very high; 7 = very low)** | |
| Tutors' technical skills | 1.19 **±**0.40 |
| Tutors' teaching skills | 1.22 **±**0.42 |
| **Ultrasound equipment (1 = satisfaction very high; 7 = very low)** | |
| Ultrasound equipment ("large") | 1.31 **±**0.55 |
| Ultrasound equipment ("pocket") | 1.55 **±**1.87 |
| **Learning outcomes assessments (1 = satisfaction very high; 7 = very low)** | |
| **Theory quizzes** | 2.18 **±**1.05 |
