# Supplementary material for: Development and evaluation of a point-of-care ocular ultrasound curriculum for medical students - a proof-of-concept study
Source: BMC Med Educ. 2023 Oct 3;23:723. doi: 10.1186/s12909-023-04723-1 (PMC10548604; doi:10.1186/s12909-023-04723-1)
Supplement: Supplementary file 6 — Supplementary Material 6 [file 12909_2023_4723_MOESM6_ESM.docx]

**Supplementary Table 1** (Development of) participant motivation; *a significance comparison of the subitems in this area was not possible because of the difference in questions asked at T1 and T2

|  | **T1** | **T2** | **p-value** |
| --- | --- | --- | --- |
|  | **Mean ±SD** | **Mean ±SD** |  |
| **Interest (1 = very high; 7 = very low)** | | | |
| Ophthalmology | 3.00 ±1.92 | 2.41 ±1.55 | 0.27 |
| Neurology | 3.36 ±1.95 | 3.19 ±1.88 | 0.64 |
| Neurosurgery | 3.61 ±1.82 | 3.48 ±1.67 | 0.85 |
| Intensive care | 3.97 ±1.67 | 3.70 ±1.49 | 0.48 |
| Emergency medicine | 3.55 ±1.86 | 3.37 ±1.64 | 0.80 |
| ENT (Ear, Nose and Throat) | 4.18 ±1.79 | 3.81 ±1.57 | 0.39 |
| **Course motivation (1 = very high; 7 = very low)** | | | |
| General motivation for the course | 1.55 ±0.75 | * | * |
| Ultrasound diagnostics (T1)/  Gain in-depth ultrasound knowledge (T2) | 1.27 ±0.45 | 1.37 ±0.57 | * |
| Ophthalmology(T1)/ophthalmologic diseases (T2) | 1.76 ±1.20 | 1.81 ±1.24 | * |
| Neurology(T1)/neurological diseases (T2) | 2.09 ±1.28 | 2.59 ±1.42 | * |
| Neurosurgery(T1)/neurosurgical diseases (T2) | 2.27 ±1.23 | 3.26 ±1.46 | * |
| Intensive care(T1)/intensive care diseases (T2) | 2.67 ±1.36 | 3.33 ±1.75 | * |
| Emergency medicine(T1)/emergency medicine diseases (T2) | 2.27 ±1.31 | 3.11 ±1.60 | * |
| ENT(T1)/ENT diseases (T2) | 2.73 ±1.61 | 3.81 ±1.94 | * |
| **Course follow-up (1 = very high; 7 = very low)** | | | |
| Gain in-depth ultrasound knowledge | * | 1.19 ±0.40 | * |
| Use of teaching medium for follow-up | * | 1.85 ±0.949 | * |
| **Attitude to ultrasound teaching (1 = fully agree ; 7 = do not agree at all)** | | | |
| Ultrasound important basic skill | 1.12 ±0.33 | 1.07 ±0.27 | 0.56 |
| Teaching of ultrasound skills during medical studies | 1.09 ±0.29 | 1.11 ±0.32 | 0.80 |
| Integration of ultrasound training into compulsory teaching | 1.24 ±0.50 | 1.26 ±0.53 | 0.93 |
| **Attitude to ultrasound media (1 = fully agree ; 7 = do not agree at all)** | | | |
| Digital teaching methods valuable addition to ultrasound education | 1.70 ±1.26 | 1.78 ±1.22 | 0.74 |
| Integration of digital teaching methods into teaching | 1.82 ±1.07 | 1.96 ±1.09 | 0.59 |
| More efficient learning with digital than with purely analog teaching media | 2.48 ±1.56 | 2.07 ±1.14 | 0.42 |
| **Attitude to teaching generally (1 = fully agree ; 7 = do not agree at all)** | | | |
| Innovative pedagogical approaches increase interest in discipline | 1.52 ±0.71 | 1.41 ±0.69 | 0.43 |
| Influence of quality of teaching in the specialty on choice of specialist medical training | 1.73 ±1.23 | 1.41 ±0.64 | 0.39 |
