# Supplementary material for: Development and evaluation of a point-of-care ocular ultrasound curriculum for medical students - a proof-of-concept study
Source: BMC Med Educ. 2023 Oct 3;23:723. doi: 10.1186/s12909-023-04723-1 (PMC10548604; doi:10.1186/s12909-023-04723-1)
Supplement: Supplementary file 7 — Supplementary Material 7 [file 12909_2023_4723_MOESM7_ESM.docx]

**Supplementary Table 3** Participants' objective (development of) competency

|  | **T1** | **T2** | **Effect size** | **p-value** |
| --- | --- | --- | --- | --- |
|  | **Mean ±SD** | **Mean ±SD** | **Cohens d** |  |
| **Total score (max. 59 points)** | 30.73 **±**11.33 | 48.00 **±**7.25 | 1.78 | <0.01 |
| Anatomical basics (max.18 points) | 8.73 **±**3.65 | 13.93 **±**2.96 | 1.55 | <0.01 |
| Ultrasound basics (max. 16 points) | 10.36 **±**5.16 | 12.63 **±**3.49 | 0.50 | 0.06 |
| Understanding of cross-sectional images (max. 10 points) | 6.72 **±**3.35 | 7.41 **±**2.82 | 0.21 | 0.58 |
| Normal findings (max. 9 points) | 3.97 **±**2.27 | 8.30 **±**0.95 | 2.40 | <0.01 |
| Pathologies (max. 6 points) | 0.94 **±**1.00 | 5.74 **±**0.59 | 5.71 | <0.01 |
